# Conflict of interest and signal interference lead to the breakdown of honest signaling

**DOI:** 10.1111/evo.12751

**Published:** 2015-09-08

**Authors:** Roman Popat, Eric J. G. Pollitt, Freya Harrison, Hardeep Naghra, Kar‐Wai Hong, Kok‐Gan Chan, Ashleigh S. Griffin, Paul Williams, Sam P. Brown, Stuart A. West, Stephen P. Diggle

**Affiliations:** ^1^ School of Life SciencesUniversity of NottinghamNottinghamNG7 2RDUnited Kingdom; ^2^ Centre for Immunity, Infection and EvolutionAshworth Laboratories, University of EdinburghKing's BuildingsWest Mains RoadEdinburghEH9 3JTUnited Kingdom; ^3^ Institute of Biological Sciences, Faculty of ScienceUniversity of Malaya50603Kuala LumpurMalaysia; ^4^ Department of ZoologyUniversity of OxfordSouth Parks RoadOxfordOX1 3PSUnited Kingdom

**Keywords:** Population structure, selection (experimental), selection (group/kin), signaling/courtship

## Abstract

Animals use signals to coordinate a wide range of behaviors, from feeding offspring to predator avoidance. This poses an evolutionary problem, because individuals could potentially signal dishonestly to coerce others into behaving in ways that benefit the signaler. Theory suggests that honest signaling is favored when individuals share a common interest and signals carry reliable information. Here, we exploit the opportunities offered by bacterial signaling to test these predictions with an experimental evolution approach. We show that: (1) reduced relatedness leads to the relative breakdown of signaling, (2) signaling breaks down by the invasion of mutants that show both reduced signaling and reduced response to signal, (3) the genetic route to signaling breakdown is variable, and (4) the addition of artificial signal, to interfere with signal information, also leads to reduced signaling. Our results provide clear support for signaling theory, but we did not find evidence for previously predicted coercion at intermediate relatedness, suggesting that mechanistic details can alter the qualitative nature of specific predictions. Furthermore, populations evolved under low relatedness caused less mortality to insect hosts, showing how signal evolution in bacterial pathogens can drive the evolution of virulence in the opposite direction to that often predicted by theory.

Animals use signals to communicate information, ranging from the need for food, to their quality as a potential mate. A problem is that individuals could potentially signal dishonestly to coerce others into behaving in a way that benefits the signaler, prompting the question of what maintains signal honesty (Dawkins 1978)? Evolutionary theory has suggested solutions to this problem, by showing how honesty can be maintained through mechanisms such as a common interest between signaler and receiver, or if dishonest signals are too costly to produce (Grafen 1990a,b; Maynard Smith and Harper 2003; Searcy and Nowicki 2005; Bradbury and Vehrencamp 2011). For example, showy ornaments can favor signal quality to mates, when only the highest quality individuals can afford to produce them (Grafen 1990a,b). Experimental and comparative studies on animals have provided support for some of the assumptions and predictions of signaling theory. For example, showing that signals are costly and condition dependent, that there can be responses to deception, and greater signaling between more related individuals (Davies 1978; Briskie et al. 1994; Agrawal et al. 2001; Kilner 2001; Cotton et al. 2004; Tibbetts and Dale 2004; Reby et al. 2005; Tibbetts and Lindsay 2008; Hinde et al. 2010; Davies 2011).

Although previous empirical research has focused on signaling in animals, the same problem of honesty has been shown to arise in the cell‐to‐cell signaling systems of bacteria (Diggle et al. 2007). This process, termed quorum sensing (QS), involves bacterial cells releasing small diffusible signal molecules. Cells respond to the uptake of these signal molecules by producing two things: (1) more signal molecule, and (2) a whole suite of extracellular factors that aid population growth, such as molecules that scavenge nutrients (Williams et al. 2007; Schuster et al. 2013). The fact that signal uptake stimulates signal production leads to a positive feedback loop, which results in a marked increase in the production of extracellular factors at high population densities. This signaling system is thought to be favored because extracellular factors can be shared more efficiently at higher population densities, and QS provides a way of coordinating their production (Darch et al. 2012). The problem of honest signaling arises because while individual cells pay the cost of producing signal molecules and the extracellular factors, the benefits of extracellular factors are shared between cells (Diggle et al. 2007; Sandoz et al. 2007). Consequently, QS could be exploited by “cheats” that avoid the costs of signaling or responding to signaling, or coerce other cells to produce more extracellular factors (Diggle et al. 2007; Sandoz et al. 2007; Kohler et al. 2009; Rumbaugh et al. 2009; Popat et al. 2012; Ghoul et al. 2014b; Pollitt et al. 2014).

Bacterial QS offers unique opportunities for experimental studies. In particular, the short generation times of bacteria mean that researchers can experimentally alter the ecological and evolutionary conditions, and then follow how the signal system evolves in response to this experimental manipulation. Progress has been made with this experimental evolution approach, by examining the relative fitness of mutants that either do not signal, or do not respond to signal, when introduced into populations of signaling individuals (Diggle et al. 2007; Rumbaugh et al. 2012; Pollitt et al. 2014). These studies have shown that mutants which do not respond to signal are able to increase in frequency in conditions of low relatedness, but not under conditions of high relatedness. However, these experiments have relied on genetic variation provided by specific defined mutants, and were run over relatively short periods of time. This results in a limited amount of genetic variation on which selection can act, and therefore only a limited phenotypic repertoire can result. In contrast, theory predicts that social competition over signaling can result in diverse strategies, including strains that coerce others to their own benefit (Brown and Johnstone 2001; Czaran and Hoekstra 2009).

Here, we utilize an alternative approach, where we start with a clonal population of the opportunistic pathogen *Pseudomonas aeruginosa*, and then examine how QS signaling evolves through the spread of de novo mutation (Sandoz et al. 2007). An advantage of this approach is that natural selection can choose from all possible mutations, and so we can test a broader range of theoretical conditions, and whether the signaling system responds to selection by changes in signaling and/or the response to signaling (Brown and Johnstone 2001; West et al. 2012; Ghoul et al. 2014b). Furthermore, mechanistic studies have previously identified a number genes involved in QS, and therefore we can sequence whole genomes and examine the repeatability of evolutionary change at the genomic level. Our aim here is to test how QS evolves in response to variation in two factors that signaling theory predicts will influence the stability of signaling systems: (1) common interest (relatedness) and (2) signal reliability.

First, theory predicts that the extent of common interest between individuals can depend upon their genetic relatedness, with a higher genetic relatedness better able to stabilize honest signaling (Grafen 1990a,b; Brown and Johnstone 2001; Maynard Smith and Harper 2003; Searcy and Nowicki 2005; Bradbury and Vehrencamp 2011). Relatedness is thought to play a key role in stabilizing signaling within families—for example, when offspring are closely related, they can be selected to altruistically reduce their rate of begging, to allow siblings with greater need to be preferentially fed (Godfray 1991, 1995). We vary genetic relatedness by dividing the population into subpopulations, and by varying the number of clones that are used to initiate each subpopulation (Griffin et al. 2004).

Second, theory predicts that if signal reliability is reduced by deceptive interference or noise, then this reduces the relative benefit/cost ratio of responding to or producing a signal, and so can lead to the breakdown of honest signaling (Maynard Smith and Harper 2003; Searcy and Nowicki 2005; Bradbury and Vehrencamp 2011). Interference or noise could potentially destabilize any form of signaling system. We test this prediction experimentally by adding synthetic QS signal to cultures, to interfere with the information provided by naturally produced signal. Finally, QS plays a key role in determining bacterial virulence in many pathogenic species (Rumbaugh et al. 2009, 2012; Pollit et al. 2014), and so in addition to examining how signaling evolved in our experiment, we examined the consequences of this for virulence using a wax moth larvae model of virulence. Our prediction is that, because a higher relatedness favors QS, this will allow *P. aeruginosa* to better exploit its host, and hence cause a higher virulence (Brown et al. 2002; West & Buckling 2003).

## Methods

### *PSEUDOMONAS AERUGINOSA* SIGNALLING SYSTEM

#### The study system

We exploit a bacterial model system to examine the evolution of signaling. The small diffusible molecules (QS molecules) comprise the signal. The response is a raft of gene regulatory changes enacted via a specialized receptor protein (Schuster et al. 2013). One such gene is the *lasB* protease that is activated in response to signal, and aids in digesting protein in the environment, leading to increased nutrient availability and reproductive (division) rate of individual cells. We have chosen to focus on this part of the QS response because (1) it allows for an experimental condition where QS endows a fitness benefit and (2) protease output can be easily measured via a biochemical assay performed on spent culture supernatants.

#### Population measurements

We measured both the aggregate behavior of diverse evolved metapopulations and the behavior of clonal populations generated by picking colonies from the diverse populations. In the case of the clonal population, we expect that each cell behaves in a similar way and so the population average is representative of the individual cell that seeded that clonal colony, all else begin equal and given minimal opportunity for new mutations to spread.

#### Honesty in bacterial signaling

The signal molecules of *P. aeruginosa* used in our experiments serve as a means to estimate population density and appropriately tune investment into extracellular enzymes that carry population density dependent benefits. An honest system therefore comprises one where a consensus signal production elicits an accurately calibrated response rule generating an appropriate level of extracellular protease output of each cell. Dishonesty can occur if individual mutants: (1) produce less signal, (2) produce more signal, or (3) respond less to signal. Such dishonesty may avoid the cost of producing either signal or the extracellular factors produced in response to signal, or to coerce other cells into producing more extracelluar factors (Brown & Johnstone 2001; West et al. 2012).

### BACTERIAL STRAINS AND GROWTH CONDITIONS

The strains we used in this study were *P. aeruginosa* PAO1 and isogenic insertion mutants in the QS genes *lasI* (PAO1∆*lasI*::Gm) and *lasRI* (PAO1∆*lasRI*::Gm, made in this study). The media we used were a rich Lysogeny broth (LB) medium (tryptone 10 g L^−1^, yeast extract 5 g L^−1^, and sodium chloride 10 g L^−1^) and a defined medium, quorum sensing medium (QSM), modified from two previous studies (Diggle et al. 2007; Sandoz et al. 2007). QSM consisted of M9 Minimal Salts, including Na_2_HPO_4_ (6.8 g L^−1^), KH_2_PO_4_ (3 g L^−1^), NaCl (0.5 g L^−1^), which was autoclaved. To this, we added the filter sterilized supplement solutions NH_4_Cl (10 mM), CaCl_2_ (0.1 mM), and MgSO_4_ (1 mM, final concentrations stated). Lastly, we added the carbon sources bovine serum albumin (BSA 1 % w/v) and CasAmino acids (CAA 0.1 % w/v) and the medium was filter sterilized. We designed the QSM medium to make maximal growth dependent upon *lasRI*‐regulated proteases. As growth proceeded, the small amount of CAA was depleted and further growth required a functional *lasRI* QS system.

### SELECTION EXPERIMENT DESIGN

**Figure 1:**
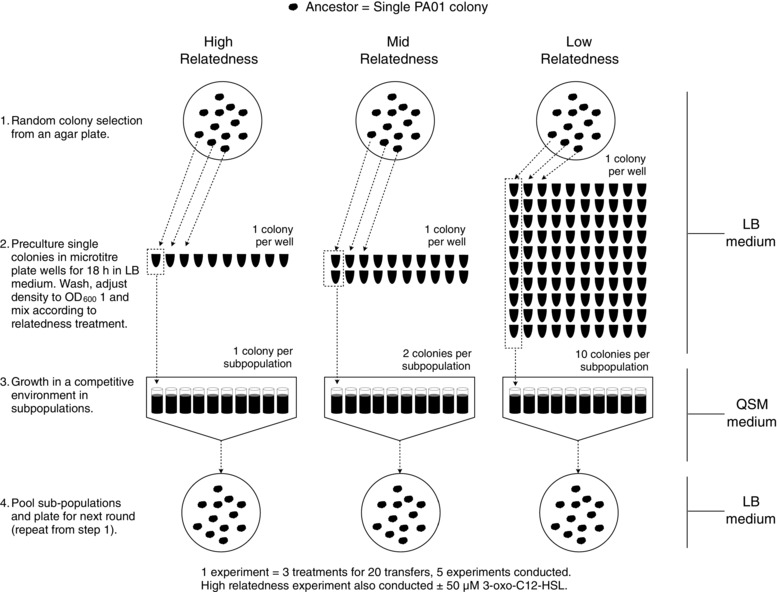
Experimental evolution regime. The experiment was initiated with a single clone of *Pseudomonas aeruginosa* PAO1. (1) Relatedness was varied by initiating each subsequent set of subpopulations with either 1 (high), 2 (mid) or 10 (low) founder clones from the previous pooled subpopulations. (2) Each founder clone was first precultured and mixed in equal density of 1, 2, or 10 founding clones. (3) We then washed and inoculated them into QSM. Each treatment therefore consisted of 10 subpopulations each with 1, 2, or 10 founding clones from the previous population. Each treatment had five independent biological replicates (total of 15 subpopulations). (4) After 24 h incubation in QSM, the 10 subpopulations were pooled and plated out to single colonies on LB agar to form the founder clones for the next round of selection. In total, 20 rounds of selection were performed. The signal addition treatment used the high relatedness regime but with the addition of 50 μM synthetic signal. Under high relatedness, clones evolving to produce less exoprotease or less QS signals will not grow to high densities in their isolated subpopulations and so will be under‐represented in successive transfers. Under low relatedness this condition is relaxed, genotypes are mixed, and clones evolving lower exoprotease production or signaling can survive selection rounds due to interactions with other genotypes in their subpopulation.

To test signalling theory, we experimentally evolved replicate populations of *P. aeruginosa* under conditions that we expected would generate differential selection for signaling. For our selection experiment, we used a *P. aeruginosa* PAO1 strain containing a chromosomal mini‐CTX*lux* fusion to the *lasI* promoter (PAO1 *lasI*::*lux*). Our experiment had four treatments: high relatedness, intermediate (mid) relatedness, low relatedness, and high relatedness with added signal. We replicated each treatment five times, giving a total of 4 × 5 = 20 selection lines (Fig. 1). Within each replicate, we subdivided each population into 10 subpopulations for each round of growth, and allowed them to grow for 24 h in a medium where the QS‐induced production of extracellular factors facilitated growth. Specifically, the QSM contained BSA, which was broken down by QS‐induced exoproteases including elastase (Diggle et al. 2007; Darch et al. 2012). We then mixed the subpopulations together before plating them out onto rich agar and picked colonies to initiate the next round of growth. This pattern of population subdivision and mixing meant that cells from tubes with higher growth were more likely to be picked into the next round of selection. As QS facilitated population growth in the medium used, this meant that QS provided a benefit at the population level, and hence there was potential for a common interest in signaling between cells (Diggle et al. 2007). We repeated this procedure for 20 rounds of growth, comprising approximately 120 bacterial generations, and then assayed our selection lines to determine how they had evolved, with respect to growth in QSM, QS signal gene (*lasI*) expression, and production of QS‐dependent extracellular protease (elastase). As we started with a single clonal PAO1 isolate, relatedness can only vary at the loci where mutation leads to genetic variation. However, because we are examining the consequences of the spread of novel mutations, this is the relatedness that matters, and in which we are interested (Hamilton 1964; Grafen 1985). We interfered with signal‐mediated communication by adding synthetic QS signal (*N*‐3‐oxo‐dodecanoyl‐l‐homoserine lactone; 3O‐C12‐HSL) to cultures. Addition of excess signal induces a maladaptively high level of exoprotease production (see Fig. S5).

### EXPERIMENTAL EVOLUTION

We initiated five replicate (5 mL) QSM cultures for each treatment (5 × 5 = 25) PAO1 *lasI*::*lux*, and incubated at 37°C for 24 h. We then diluted these cultures and plated out to single colonies. We inoculated the resulting colonies into 300 μL LB cultures and incubated at 37°C for 18 h. We used these LB cultures to initiate subsequent QSM cultures in the following way (Fig. 1). Each replicate QSM population consisted of 10 subpopulations. We initiated each subpopulation according to treatment (high relatedness = 1 colony, mid relatedness = 2 colonies, low relatedness = 10 colonies), after correcting for optical density at 600nm (OD_600_) and washing inoculating cells in fresh QSM. We propagated signal supplementation treatments using the high relatedness regime, but in the presence of 50 μM of the signal molecule *N*‐(3‐oxododecanoyl)‐l‐homoserine lactone (3‐oxo‐C12‐HSL) synthesized as described before (Chhabra et al. 2003). We then incubated these subpopulations at 37°C for 24 h, after which we pooled within treatments, diluted and plated to single colonies (Griffin et al. 2004). We used these colonies to initiate the subsequent round and continued the procedure for a total of 20 transfers. We froze aliquots of evolved populations after pooling in 20% glycerol at −80°C. In all of the assays that followed experimental evolution, we revived frozen cultures of the evolved lines and the ancestral line and assayed the behaviors side by side. This allows for a direct comparison of the ancestral and evolved behaviors.

### PHENOTYPIC ASSAYS

Following experimental evolution, we analyzed the phenotypes of the resulting evolved populations to understand which conditions generated changes in signaling and response behaviors. We inoculated 5 μL of the frozen stock of each population into 5 mL of sterile LB and incubated at 37°C for 18 h with shaking. We then treated these cultures in three different ways to separately assess (1) growth in QSM, (2) exoprotease production, and (3) *lasI* expression in the following ways. For growth in QSM, we used sterile QSM to wash and correct cultures to OD_600_ 1.0 and inoculated these into 30 μL into 300 μL sterile QSM in a microplate. We then incubated microplates in a Tecan™ plate reader at 37°C and measured culture density (OD_600_) every hour for 24 h and reported growth after 24 h. For exoprotease production, we measured elastolytic activity of bacterial culture‐free supernatants (passed through a 0.22 μm pore filter) by using the elastin Congo red (ECR, Sigma Gillingham, Dorset, UK) assay (Ohman et al. 1980). We added a 100 μL aliquot of bacterial supernatant to 900 μL ECR buffer (100 mM Tris, 1 mM CaCl_2_, pH 7.5), containing 20 mg ECR and incubated with shaking at 37°C for 3 h. We removed insoluble ECR by centrifugation and we measured the absorption of the supernatant at 495 nm. We used LB medium as a negative control. For signal gene expression, we assessed *lasI* signal gene expression by measuring light output from cultures. Using sterile LB, we washed and corrected cultures to OD_600_ 0.1 in 300 μL in a microplate. We then incubated microplates in a Tecan™ plate reader at 37°C and measured culture density (OD_600_) and relative light units (RLUs) every hour for 6 h and reported the ratio between light output and culture density (RLU/OD_600_) at its peak expression (3 h). To verify that signal gene expression of evolved populations reflected the amount of signal molecule produced, we correlated measurements of signal molecule concentration in supernatants with measurements of signal gene expression (Fig. S1).

### DETERMINING RELATIVE FITNESS

Following experimental evolution, we measured the fitness of resulting populations and individuals. We assessed the relative fitness of PAO1 ∆*lasIR* in the presence of the PAO1 WT by coculturing the two strains. We labeled the *lasIR* mutant with a chromosomal insertion of a promotorless mini‐CTX*lux* cassette to distinguish between the two strains using light detection. We inoculated a single colony of each separately into 5 mL of sterile LB medium and incubated for 18 h at 37°C with shaking. We then washed and corrected these cultures to OD_600_ 1.0 in sterile QSM and mixed these in the ratio 95:5 (WT:mutant). We then used this mixture to inoculate 3 μL into 10 replicate 300 μL cultures in both the presence and absence of 50 μM 3‐oxo‐C12‐HSL signal molecule and incubated these cultures at 37°C for 24 h. We determined WT and mutant frequencies by plating to single colonies on sterile LB agar, and assessing 200 colonies for light output using a Hamamatsu light camera. We calculated mutant relative fitness (*w*) using the formula [*x*_2_(1 − *x*_1_)]/[*x*_1_(1 − *x*_2_)], where *x*_1_ is the initial proportion of cheats in the population and *x*_2_ is their final proportion (Ross‐Gillespie et al. 2007). For example, *w* = 2 would correspond to the mutant growing twice as fast as the wild‐type cooperator.

### DETERMINING THE VIRULENCE OF POPULATIONS

QS signaling regulates the damage to hosts, termed virulence, in *P. aeruginosa* and a number of other pathogenic species (Rumbaugh et al. 2009, 2012). Consequently, we tested whether our different treatments also altered virulence. We measured the virulence of the evolved populations using a filtered supernatant assay injected into the larvae of the greater wax moth (*Galleria mellonella*). We inoculated 5 μL of the frozen stock of each evolved population into 5 mL of sterile LB and incubated at 37°C for 16 h with shaking. We then centrifuged cultures at 9500 rpm for 3 min before passing the supernatant through a 0.2 μm pore filter, leaving a cell‐free supernatant. For each supernatant, we injected a group of 30 greater wax moth larvae, with 50 μL of sterile supernatant between the hind pair of prolegs using a U‐100 (29G) insulin syringe (SLS) attached to a Tridak Stepper™ for accurate dispensation. We then placed the larvae in pill box compartments (15 × 2 × 2 cm) to keep them separate. After 1 h at room temperature, we assessed virulence using two indicators: death and hemolymph loss. We judged a wax worm to be dead if it did not respond to external stimuli and we judged hemolymph loss to have occurred if the wax worm had lost enough hemolymph to cover the base of its compartment. As controls, we tested WT and *lasR* mutant supernatant and LB media on separate wax worm batches.

### SEQUENCING LIBRARY PREPARATION AND SEQUENCING

To determine the underlying genetic changes responsible for phenotypic changes in signaling and response behaviors, we sequenced the genomes of a randomly selected subset of evolved individual clones. We prepared sequencing libraries using the Nextera DNA sample preparation kit and indexed using the Nextera index kit (Illumina, Nextra, San Diego, CA) according to the manufacturer's recommendation. Briefly, we tagged 50 ng of genomic DNA and fragmented in the presence of transposons with adapters. We purified and enriched fragmented DNA via limited‐cycle PCR. We purified the resulting sample libraries and evaluated the quantity and quality of the libraries using a KAPA library quantification kit (Kapa Biosystems, MA) and High Sensitivity DNA Kit (Agilent Technologies, CA). We pair end sequenced each library (2 × 250 bp) using a MiSeq Personal Sequencer (Illumina).

### MEASURING 3‐OXO‐C12‐HSL CONCENTRATIONS

To assess whether expression of the signal production gene (*lasI*) served as an accurate measure of signal production, we also measured signal concentrations in spent supernatants of evolved clones. Populations were inoculated into LB at an initial turbidity of OD600 0.01 and incubated for 8 h at 37°C and 200 rpm. Cultures were then centrifuged and filtered to remove cells. Cell‐free supernatants were diluted 1:100 and then mixed 1:1 with a log phase culture of an *Escherichia coli* bioreporter. This mixture was incubated for 3 h and then luminescence recorded. To estimate 3‐oxo‐C12‐HSL concentration, the luminescence of unknown samples was compared to that when given a range of known concentrations.

### STATISTICAL ANALYSES

**Figure 2:**
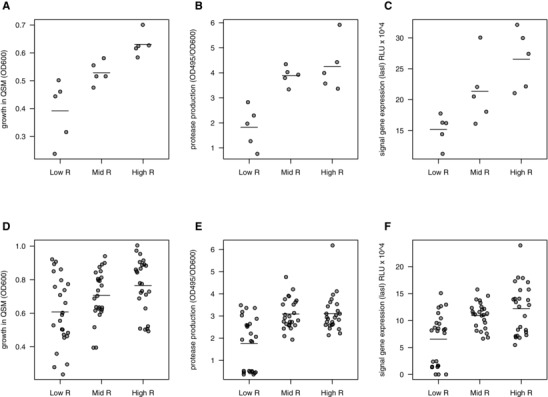
Relatedness and common interest. A lower relatedness led to the evolution of reduced mean levels of: (A) growth in QSM; (B) exoprotease (elastase) production, and (C) signal gene (*lasI*) expression at the level of the population. Each point represents a replicate evolutionary line (five replicates) and the bars represent the means of each group. The horizontal bars represent the means. We then analyzed the same phenotypes but from five individual colonies isolated from each replicate population (5 × 15 = 75 colonies). We found the same general pattern as at the population level that a lower relatedness led to the evolution of reduced mean levels of: (D) growth in QSM, (E) exoprotease (elastase) production, and (F) signal gene (*lasI*) expression (see also Fig. S3 and S4). Each point in D–F represents the value for a single clone. The horizontal bars represent the means.

We performed all statistical analyses and data visualizations using the open source statistical platform R (Ihaka and Gentleman 1996) version 2.14.0 in particular implementing the packages reshape (Wickham 2007) and *nlme*. We analyzed the three phenotypes; growth in QSM, exoprotease production, and signal gene expression, and within populations variance of these three phenotypes by one‐way analysis of variance (ANOVA). To test an ordered null hypothesis between the relatedness treatments, we used the ordered heterogeneity test (Rice and Gaines 1994), combining the F statistic from ANOVA with Spearman's rank correlation coefficient. Because individual clones were assayed in experimental blocks, we analyzed the phenotypes of individuals using a mixed effects model with experimental block as the random factor. In this case, *P* values are estimated and so the OH test was not used; however, model coefficients were always ordered from high to low relatedness recovering the same pattern as at the population level (Fig. 2D–F). Experiments where signal was added were analyzed using Mann–Whitney *U* tests. We analyzed relative fitness of a mutant via one and two sample (Welch) *t* tests. We analyzed killing and hemolymph loss of insect hosts by generalized linear mixed model with a Poisson error distribution and block as a grouping factor. We checked the assumptions of all models used.

## Results

### COMMON INTEREST AND RELATEDNESS

As predicted by signaling theory, in our selection experiment (Fig. 1), we found that the reduction in common interest between interacting individuals, caused by a lower relatedness, led to a relative breakdown of the QS signaling system. Analyzing the aggregate behavior of whole populations, we found a positive relationship between relatedness and: (1) growth in QSM medium (Fig. 2A: OH test rsPc = 0.925, *P* < 0.001), (2) exoprotease production (Fig. 2B: OH test rsPc = 0.755, *P* < 0.01), and (3) expression of the *lasI* gene that is involved in 3‐oxo‐C12‐HSL signal production (Fig. 2C: OH test rsPc = 0.789, *P* < 0.01). We found the same positive relationship between signaling and relatedness when analyzing individual clones from the evolved populations (Fig. 2D–F: linear mixed effects models, growth in QSM medium; *F*_3,68_ = 8.16, *P* = 1 ×10^−4^, exoprotease production; *F*_3,68_ = 26.2, *P* < 0.0001, *lasI* expression; *F*_3,68_ = 21.6, *P* < 0.0001).

Signaling theory further predicts that when relatedness is lower, this breakdown of signaling is caused by cheats who exploit the cooperative signaling of others (Ghoul et al. 2014b). We therefore predicted that populations would contain a wider variety of phenotypes with decreasing relatedness—some cooperators, some cheats. Consistent with this, we found that both growth and signal gene expression were more variable in the lower relatedness treatment relative to the others (Fig. S2A, C: *F*_1,13_ = 10.9, 12.2, *P* = 0.002, 0.004). Although we found the same pattern with exoprotease production, the difference in variance was nonsignificant, possibly due to one outlying replicate with a particularly low variance (Fig. S2B: *F*_1,13_ = 2.96, *P* = 0.109).

**Figure 3:**
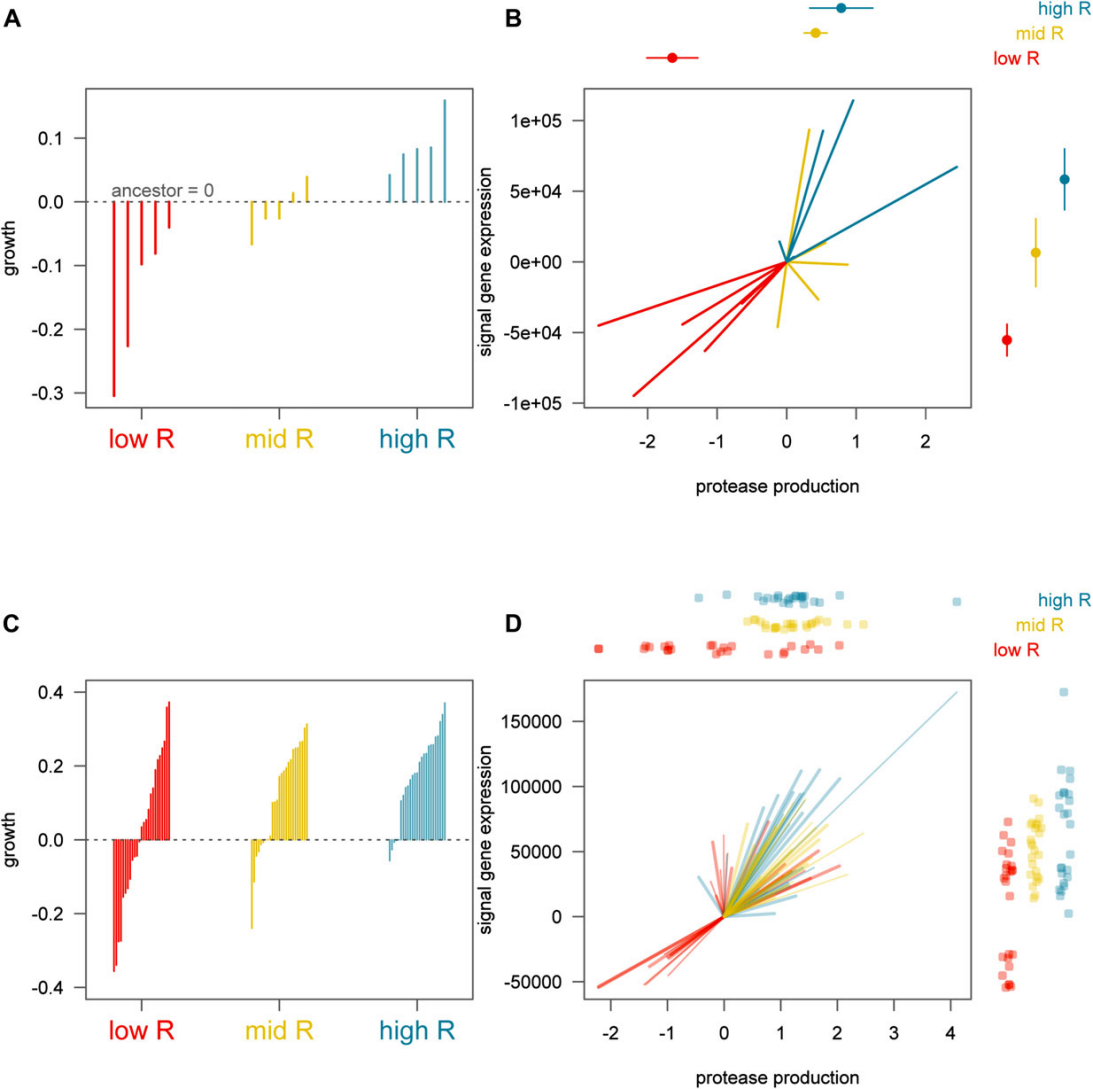
Evolutionary change from the ancestor is bidirectional. (A) Fitness of populations evolved under low, mid, and high relatedness. The dotted line at 0 represents the ancestral value. Each line emanating from the ancestral value represents a replicate metapopulation assayed as a whole (B) Evolved phenotypes show a reduction in both signal production and cooperation in low relatedness, no significant change in mid relatedness, and an increase from ancestor in high relatedness treatments. The coordinates (0, 0) represent the ancestral values. Each line emanating from the ancestral value represents a replicate metapopulation (C–D). The same general pattern is observed when analyzing the phenotypes of individual clones drawn randomly from each metapopulation. (C) The change in fitness from ancestor of clones is negative in low relatedness, nonsignificant in mid relatedness, and positive in high relatedness. Each line emanating from the ancestral value represents a single clone drawn at random from a metapopulation. (D) Evolved phenotypes of individual clones also show a reduction in both signal production and cooperation in low relatedness, no significant change in mid relatedness, and an increase from ancestor in high relatedness treatments.

Our experimental manipulation of relatedness also leads to a difference in effective population size across treatments, with a lower relatedness corresponding to a larger effective population size, which makes natural selection more efficient. Consequently, an alternative explanation for our observed relationship between signaling and relatedness would be if signaling and cooperation are being selected against in all treatments, but they are being lost more rapidly in the low relatedness treatment where effective population sizes are larger. However, this alternative hypothesis is not supported by the fact that the positive relationship between relatedness and signaling was due to both increased signaling at high relatedness and reduced signaling at low relatedness (Fig. 3). When analyzing populations (Fig. 3A, B), the change in fitness from the ancestor is negative in low relatedness (*t*_3,12_ = −4.70, *P* = 0.0005), nonsignificant at mid relatedness (*t*_3,12_ = 1.19, *P* = 0.26), and positive in high relatedness (*t*_3,12_ = 2.25, *P* = 0.044), respectively. The same is true for exoprotease production and signal gene (*lasI*) expression, where the change from ancestor is negative in low relatedness, not significantly different in mid relatedness, and increased in high relatedness treatments. (Protease: *t*_3,12_ = −4.62, −0.41, and 2.73; *P* = 0.0006, 0.69, and 0.018. Signal expression: *t*_3,12_ = −2.802, 0.329, and 2.958; *P* = 0.016, 0.75, and 0.012, respectively.) This bidirectional change was also observed when analyzing the evolved fitness and phenotypes of individual clones (Fig. 3C, D). The change in fitness from the ancestral value is negative in low relatedness (*t* = −3.64, *P* < 0.001), nonsignificant in mid relatedness (*t* = −1.34, *P* = 0.18), and positive in high relatedness (*t* = 4.38, *P* < 0.001). Evolved phenotypes of individual clones also show a reduction in both signal production and cooperation in low relatedness, no significant change in mid relatedness, and an increase from ancestor in high relatedness treatments. (Protease: *t* = −5.41, −0.07, and 6.49; *P* < 0.001, = 0.94, and < 0.001. Signal expression: *t* = −6.86, −1.52, and 5.15; *P* = < 0.001, = 0.13, and < 0.001, respectively.)

### SIGNALING AND COERCION

The relative breakdown of signaling (reduced signal and response) could have occurred via decreased production of signal, or decreased response to signal. Signaling theory developed specifically for QS predicts that a lower relatedness will lead to: (1) a reduced response to signaling, and (2) signal production showing a domed relationship with relatedness (Brown and Johnstone 2001). The reason for this domed relationship is that as relatedness is reduced from that in clonal populations, individuals are initially selected to “coerce” other individuals into producing more extracellular factors, while producing less themselves, until relatedness becomes so low, that both signaling and responding are disfavored.

**Figure 4:**
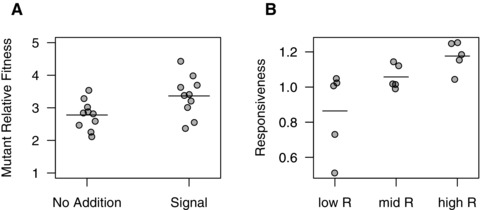
Coercion and responsiveness to signal. (A) The fitness of a rare QS mutant that does not respond to signal, relative to a PAO1 wild‐type with a fully functioning QS system, when grown in a mixed culture (inoculated at a ratio of 95:5, WT:mutant). The mutant grows faster than the wild‐type (as shown by fitness >1), and this difference is further increased by the addition of synthetic signal. (B) A lower relatedness led to a lower response to signal, as measured by *lasI::lux* expression in the presence versus absence of synthetic signal. Each point represents a replicate evolutionary line and the bars represent the means of each group.

We tested whether coercion is possible, using our un‐evolved PAO1 strain, which has a fully functional QS system, and a *lasIR* mutant (PAO1∆*lasRI*::Gm) that does not respond to or produce signal, and which therefore does not produce exoprotease or other extracellular factors. When grown together, the mutant had a higher relative fitness, because it benefited from the proteases produced by PAO1, without paying the cost of producing them (Fig. 4A: *t*_9_ = 12.75, *P* < 0.001). When we added 3‐oxo‐C12‐HSL signal, to simulate the mutant coercing PAO1 into producing more protease, this further increased the relative fitness of the mutant (Fig. 4A: *t*_16.2_ = 2.43, *P* < 0.05). This shows that, the combination of a higher level of signal production and a reduced response to signal could potentially provide a fitness benefit by coercing other cells into cooperatively producing extracellular factors at a greater rate and therefore such coercing phenotypes could be expected to evolve within populations.

We found however, in our experimental evolution study, that a lower relatedness led to a reduction in both the production of and response to signal, with no evidence of coercion at intermediate relatedness. As described above, populations evolved at lower relatedness showed a reduction in both exoprotease production (Fig. 2B, E: OH test rsPc = 0.755, *P* < 0.01) and signal gene expression (Fig. 2C, F: OH test rsPc = 0.789, *P* < 0.01). The reduced exoprotease production could have been caused purely by reduced signaling, or could have also been due to a reduced response to signal. We tested for a reduced response to signal by measuring signal gene expression with and without the addition of synthetic signal. We found that the lines evolved at a lower relatedness showed a reduced response to the addition of signal (Fig. 4B: OH Test rsPc = 0.722, *P* < 0.01).

To examine how changes at the genomic level correlated with signaling, we sequenc the whole genomes of three individual isolates from each of the relatedness populations (15 populations × 3 = 45 isolates). We found a total of 47 genomic loci containing single nucleotide polymorphisms (SNPs) across all the sequenced individuals, and the total number of SNPs was 139 (Table S1). Although the number of SNPs did not vary between treatments (Fig. S3A: OH test rsPc = 0.697, *P* > 0.05), we only observed nonsynonymous SNPs known to influence QS (*lasI*, *rsaL*, and *vfr*) in isolates from our low relatedness populations (Fig. S3B). Each of these three QS‐linked mutations was found in only one of the populations, but always in multiple clones within that population (Fig. S4).

### SIGNAL INTERFERENCE

**Figure 5:**
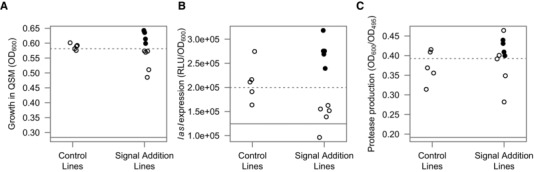
Signal interference. Populations were evolved in media containing 50 μM synthetic signal (signal interference), and then assayed with and without signal addition. Signal interference led to the evolution of: (A) a lower fitness in a QS‐requiring medium that could be restored with signal, (B) a reduced level of signal gene expression that could be restored with signal, and (C) unchanged level of exoprotease production. Reduced signal expression but unchanged exoprotease production suggests that interference with the information provided by signal molecules can lead to reduced selection for signaling. Each point represents a replicate evolutionary line, the open circles represent no addition of synthetic signal and the closed circles represent cultures with addition of 50 μM synthetic signal. The dashed lines represent the means of the unevolved PAO1 wild‐type with a fully functioning QS system, and the solid lines represent a ∆*lasIR* mutant that does not produce or respond to synthetic signal.

We added 50 μM of 3‐oxo‐C12‐HSL signal to the cultures in the signal interference treatment, to interfere with the information that signal concentration provides about cell density. In this case, we would expect a nonoptimal response to signal, and a subsequent reduction in population growth (Fig. S5). Consequently, theory predicts that the signaling system should respond to this, by evolving either a lower signal production and/or lower response to signal. We found support for this prediction (Fig. 5). First, our lines which had been evolved in the presence of synthetic signal, grew to a higher density than the controls when signal was added (Mann–Whitney *U* = 25, *P* < 0.01), but a lower density than the controls when the signal was not added (Fig. 5A; Mann–Whitney *U* = 0, *P* < 0.01). This suggests they have evolved to take account of the additional signal in the culture.

Second, evolution in the presence of synthetic signal led to selection for reduced signaling. When grown without synthetic signal, our lines, which had been evolved in the presence of synthetic signal, showed lower expression of the *lasI* signal production gene than the control lines (Fig. 5B; Mann–Whitney *U* = 0, *P* < 0.01). When signal was added to the lines evolved in the presence of signal, their expression of the *lasI* signal gene was significantly higher than the controls (Fig. 5B; Mann–Whitney *U* = 25, *P* < 0.05), again suggesting compensation to allow for the artificial signal being added. A possible reason for the selection on signal production in our experiment is the consequences for protease production. However, we found that protease production did not differ between lines evolved in the presence and absence of signal (Fig. 5C; Mann–Whitney *U* = 12, *P* > 0.05), and that signal addition did not significantly increase the production of protease in lines evolved with the addition of signal (Fig. 5C; Mann–Whitney *U* = 19, *P* > 0.05).

### SIGNALING AND PATHOGEN VIRULENCE

**Figure 6:**
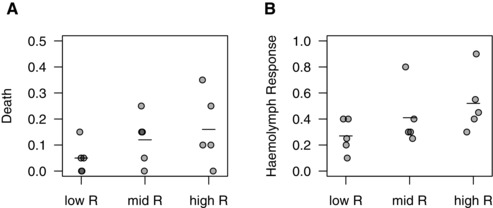
Signaling and virulence in wax moths. A lower relatedness led to the evolution of: (A) reduced mortality rate of wax moth larvae, and (B) reduced occurrence of hemolymph loss (indicative of reduced tissue damage). Each point represents a replicate evolutionary line, and the lines reveal the blocking design of the virulence experiments. Data are plotted as proportion of wax moth larvae killed (A) or incurring hemolymph loss (B).

The extracellular factors produced in response to QS by pathogenic species play key roles in population growth within hosts, and so are major determinants of the damage to the host (Rumbaugh et al. 2009; Pollitt et al. 2014). Indeed, many are referred to as “virulence factors.” Consequently, anything which causes variation in parameters such as the common interest between cells, and which will therefore influence the nature of QS, could also influence the evolution of virulence. We tested this by injecting larvae of the greater wax moth (*G. mellonella*) with cell‐free culture supernatants from our evolved populations. We used supernatants, as many of the QS‐dependent virulence factors produced by *P. aeruginosa* function extracellularly, and so this assay is used to measure extracellular toxin‐mediated virulence (Hossain et al. 2006). We found that the populations evolved under lower relatedness led to significantly lower rates of both host death (Fig. 6A: *z* = −2.18, *P* = 0.029) and reduced occurrence of hemolymph loss, which is indicative of reduced tissue damage (Fig. 6B: *z* = −2.74, *P* = 0.006).

## Discussion

We used QS in the opportunistic pathogen *P. aeruginosa* to carry out an experimental evolution study of signaling (Fig. 1). We found that: (1) a reduced relatedness led to the relative breakdown of signaling within populations due to the invasion of mutants that showed a reduction in both signaling and response to signaling (Figs. 2 and 3); (2) signaling mutants harbored diverse genetic mutations, some of which were in the described QS regulatory network (Fig. S3 and S4); (3) the addition of artificial signal, to interfere with the information that signal concentration provides about cell density, led to selection for reduced signaling (Fig. 5); and (4) the breakdown of signaling led to reduced virulence in wax moths (Fig. 6). In contrast, while coercion of other cells to cooperate at a higher rate is possible (Fig. 4), we found no evidence for coercion in populations evolved at low or intermediate relatedness.

Overall, the consequences of manipulating relatedness provide clear support for the general prediction that a common interest between interacting individuals can help maintain honest signaling. This prediction has been made in a number of theoretical models, including models of QS, offspring begging for food from parents, and the Philip Sidney game (Grafen 1990a,b; Maynard Smith and Harper 2003; Searcy and Nowicki 2005; Bradbury and Vehrencamp 2011). Relatedness matters, because a lower relatedness means that signalers and signaling cheats will be able to interact, such that the cheats can exploit the signalers. We and others have previously found support for this prediction, with short term selection experiments that introduced mutants that did not respond to signal into populations of signaling individuals (Diggle et al. 2007; Rumbaugh et al. 2012; Pollitt et al. 2014). Here, we have expanded upon this work by examining selection on novel genetic mutations. This allows natural selection to choose from all possible mutations, and so we have been able to examine how both signaling and the response to signaling evolve at different relatedness. We found that cheats with both reduced signaling and reduced response to signaling were able to increase in frequency in our lower relatedness treatments.

Theory predicts that the level of signaling should go up and then down as relatedness is reduced, because intermediate relatedness selects for individuals to “coerce” other individuals into producing more extracellular factors (Brown and Johnstone 2001). In contrast to this predicted domed relationship, we found that the level of signaling showed a monotonically decreasing relationship with decreasing relatedness (Fig. 2C, F). A possible explanation for this discrepancy is that previous theory treated signal production and response as independently evolving traits, whereas the process of positive autoregulation introduces a positive coupling between signal production and response—limiting the rapid evolution of coercive high‐signal, low‐response strategies. This emphasizes that while signaling theory provides a general explanation for numerous forms of communication, mechanistic details can alter even the qualitative nature of specific predictions. We suggest that variation in the mechanistic or life history details across species may help resolve other current controversies, such as the function of offspring begging (Mock and Dugas 2011).

When we examined genomic changes in individuals taken from populations, we observed nonsynonymous SNPs known to influence QS (*lasI*, *rsaL*, and *vfr*) in isolates from our low relatedness populations (Figs. S3 and S4). These three loci are all known to influence QS and signaling in *P. aeruginosa* (Schuster et al. 2013), and help to explain the breakdown of signaling we observed in the low relatedness populations. The *lasI* gene regulates the synthesis of the 3‐oxo‐C12‐HSL signal molecule, and so mutations in this gene lead to a loss of signal production. Vfr is a cyclic adenosine monophosphate (AMP) receptor protein (CRP) homolog and binds to a CRP‐binding consensus sequence upstream of the *lasR* gene (Albus et al. 1997). Consequently, Vfr is required for full expression of *lasR*, and a *vfr* mutant would be expected to respond less well to signal than a wild‐type cell. RsaL is a repressor of QS in *P. aeruginosa*. Whole gene deletions of *rsaL* have previously been shown to increase transcription of the *lasI* signal synthase gene, and enhance QS signal production (Rampioni et al. 2007). Therefore, loss of RsaL function in our mutants should have resulted in upregulation of *lasI* and signal production, but this is not what we observed. This suggests that the mutation in *rsaL* identified in our study, may have led to an enhanced RsaL activity, which further dampened, rather than enhanced, the production of signal.

As signals become less reliable there should be an increasing selection to not respond to them (Maynard Smith and Harper 2003; Searcy and Nowicki 2005). We tested this by performing selection experiments in artificially high concentrations of 3‐oxo‐C12‐HSL, thus distorting the informational content of the signal. We found that cells in the signal addition line evolved to take account of the additional signal in the culture, with a lower expression of the *lasI* gene, and hence reduced signal production (Fig. 5). A possible reason for the selection on signal production is that the addition of artificial signal led to a costly overproduction of proteases, resulting in selection to reduce their production, by either reduced signaling, or reduced response to signaling. However, we found that protease production did not differ between lines evolved in the presence and absence of signal and that signal addition did not significantly increase the production of protease in lines evolved with the addition of signal. This unexpected result suggests that there is a cost of excess signal molecule independent of the increased protease output, and provides another example of where mechanistic details appear to influence the evolutionary outcome.

Finally, we also investigated the virulence consequences of the evolution of the QS system in the different treatments. QS plays a major role in the virulence of pathogenic bacteria such as *P. aeruginosa*, and so we predicted that a higher relatedness would allow more cooperative exploitation of the host, and hence a higher relatedness also influence the evolution of virulence (Brown et al. 2002; West & Buckling 2003). We found support for this prediction, with a higher relatedness leading to a higher virulence (Fig. 6). This has two implications for our understanding of pathogenic virulence. First, it is commonly assumed that a lower relatedness (higher strain diversity) should select for a higher virulence in parasites, because the competition for host resources selects for higher growth rates (Frank 1996). However, there is a relative lack of empirical support for this theoretical prediction (Herre 1993; Read and Taylor 2001). Our results show that one explanation for this is that higher relatedness favors signaling, which leads to greater growth and hence higher virulence, giving the opposite prediction (West and Buckling 2003). Second, our results illustrate how the evolution of the QS signaling system could influence or be exploited as part of a medical or veterinary intervention strategy. For example, any intervention that leads to a lower (or higher) relatedness between interacting cells would select for lower (or higher) virulence. Furthermore, the introduction into existing infections of mutants that did not either signal or respond to signal could be exploited as a way to either reduce virulence or hitch‐hike medically beneficial genes into populations, such as antibiotic susceptibility, or QS signal degrading enzymes, such as AiiA (Dong et al. 2000; Brown et al. 2009).

## Supporting information


**Figure S1**. *lasI* expression is indicative of signal production.
**Figure S2**. The within population variance in QS phenotypes increases in low relatedness treatments.
**Figure S3**. Mutations and selection with varying relatedness.
**Figure S4**. Phenotypes of individual clones are explained by mutations in key QS regulators.
**Figure S5**. Varying the cost:benefit ratio of signaling.

**Table S1**. List of SNPs from individual isolates.
