# Supplementary material for: Conflict of interest and signal interference lead to the breakdown of honest signaling
Source: Evolution. 2015 Sep 8;69(9):2371–83. doi: 10.1111/evo.12751 (PMC4862024; doi:10.1111/evo.12751)
Supplement: Supplementary file 1 — Figure S1. lasI expression is indicative of signal production. Figure S2. The within population variance in QS phenotypes increases in low relatedness treatments. Figure S3. Mutations and selection with varying relatedness. Figure S4. Phenotypes of individual clones are explained by mutations in key QS regulators. Figure S5. Varying the cost:benefit ratio of signaling. [file EVO-69-2371-s001.pdf]

Conflict of interest and signal interference lead to the breakdown of honest signalling

Roman Popat, Eric J. G. Pollitt, Freya Harrison, Hardeep Naghra, Chan Kok Gan, Ashleigh S. Griffin, Paul Williams, Sam P. Brown, Stuart A. West, Stephen P. Diggle

Supplementary Information

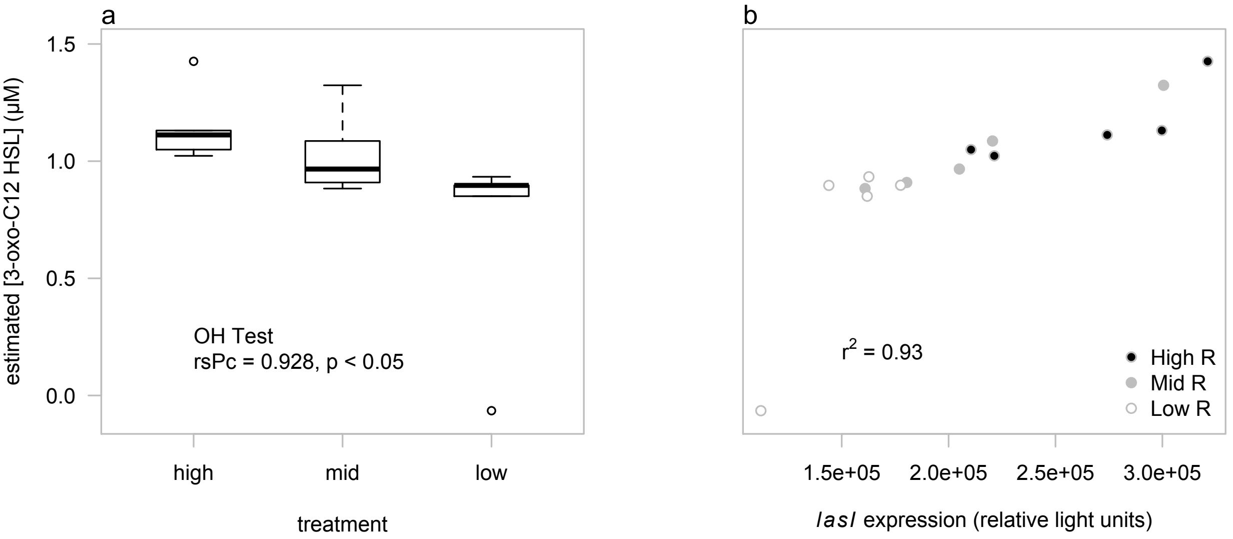

Figure S1. *lasI expression is indicative of signal production.* Concentrations of 3-oxo-C12-HSL in evolved populations decreases with decreasing relatedness (a. OH test, rsPc = 0.928, $p < 0.05$) indicating that populations evolved under lower relatedness evolve a lower overall production of 3-oxo-C12-HSL. Measurements of *lasI::lux* expression correlate well with concentrations of 3-oxo-C12-HSL except at very low concentrations of 3-oxo-C12-HSL (b. $r^2 = 0.93$), indicating that *lasI::lux* is a robust indicator of signal production. 3-oxo-C12-HSL concentrations were determined by mixing cell free supernatants with a luminescing *E. coli* reporter strain and calibrating the luminescence of the reporter with known concentrations.

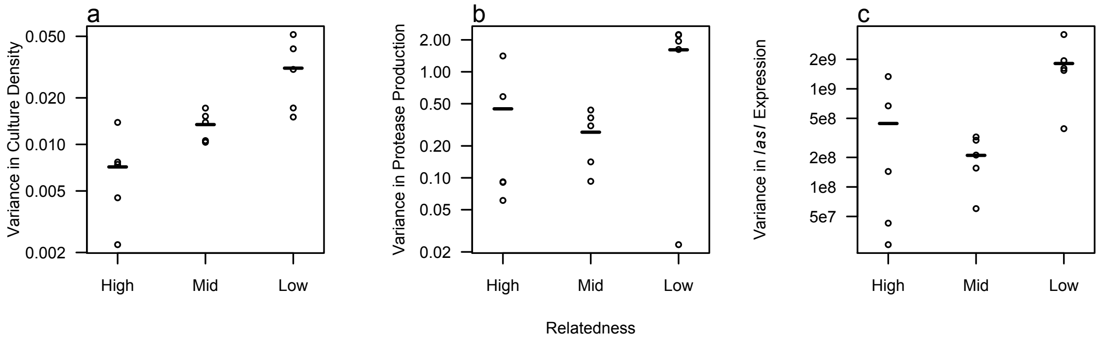

Figure S2: The within population variance in QS phenotypes increases in low relatedness treatments. We found that growth and signal gene expression were more variable in the lower relatedness treatment relative to the others (A, C; $F_{1,13}$ = 10.9, 12.2, p = 0.002, 0.004). Although we found the same pattern with the variance in exoprotease production, it was non-significant, possibly due to one outlying replicate which had a particularly low variance (Fig. 1b; $F_{1,13}$ = 2.96, p = 0.109). This increase in variance is often driven by the appearance of mutants that do not produce signal or protease and therefore also do not grow to high density.

a

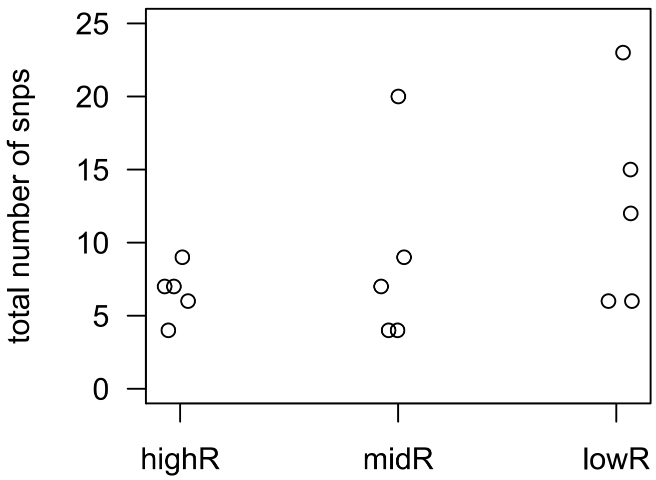

b

	high R	mid R	low R
Hypothetical proteins	21	23	29
Enzymes	8	8	11
Secretion	2	4	3
Transcriptional Regulation	0	3	6
RNA	1	1	4
Sensor	0	1	5
Quorum sensing	0	0	3
Cell shape	0	2	0
Efflux	1	1	0
Iron transport	0	1	1

Figure S3: Mutations and selection with varying relatedness (a) The number of SNPs in each population does not differ significantly with relatedness (OH test rSPC = 0.697, $p > 0.05$). This leads to the conclusion that the mutational supply either does not differ between treatments or does not lead to a significant difference in mutations across our treatments. Each point represents the total number of SNPs in three randomly chosen individuals from a single population. (b) Certain functional classes of genes are mutated to a greater degree as relatedness declines. The numbers in the table represent the sum of SNPs found in 3 individuals from each of 5 populations for each treatment. SNPs are found in a variety of different types of genes, but most notably, mutations known to be directly involved in quorum sensing are only found in the low relatedness treatment.

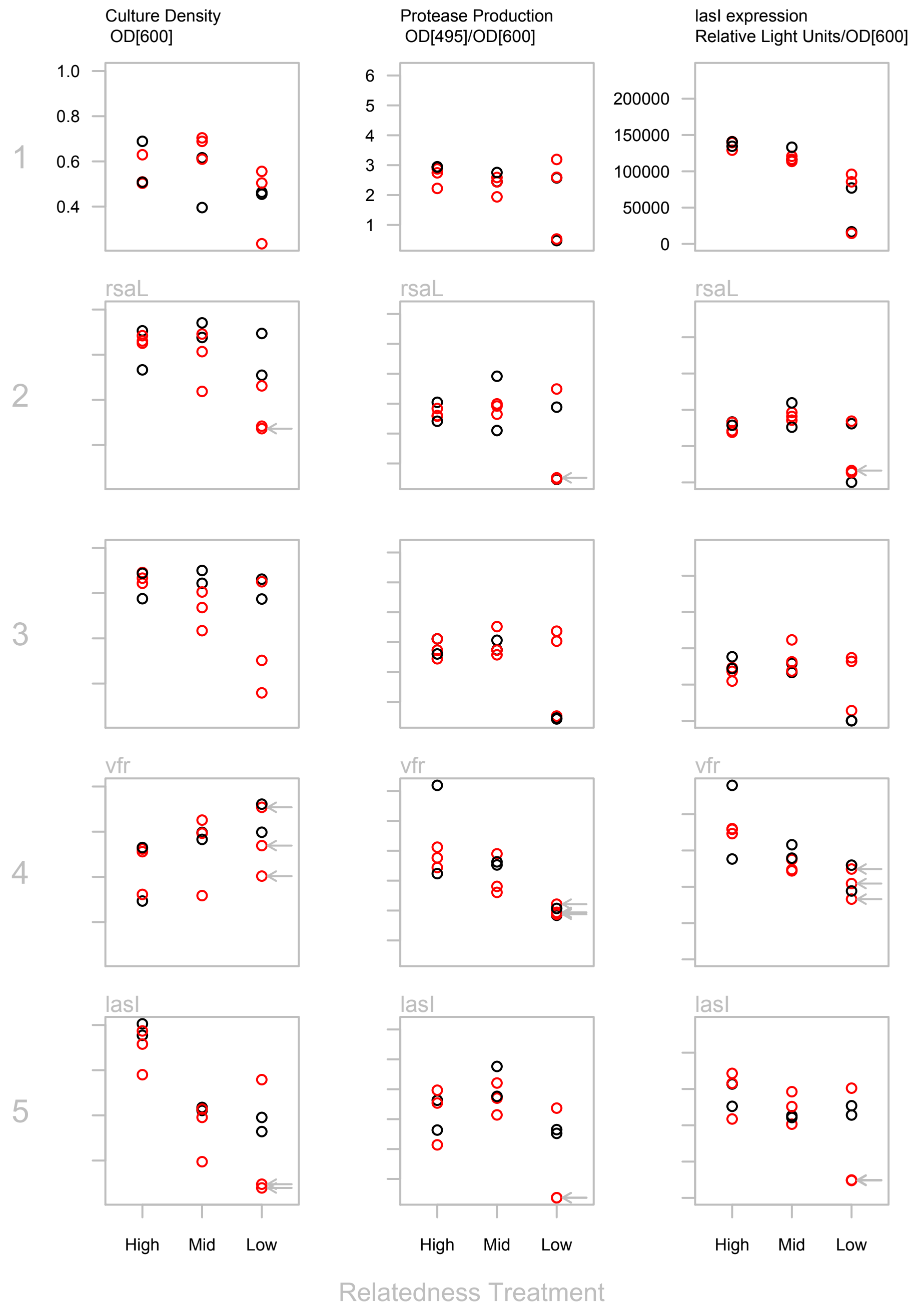

Figure S4. *Phenotypes of individual clones are explained by mutations in key QS regulators.* We measured phenotypic and genotypic changes after evolution in 5 clones of each replicate (1 - 5). Growth in QSM (left), protease production (middle) and *lasI* expression (right) were all measured in the same way as described for the population level (see Materials and Methods). The x axes represent the relatedness treatment. The scale on the y axes is constant in each collumn. Each row is a replicate of the entire experiment. Each data point represents a single clone isolated from the pooled metapopulation of the replicate/treatment combination as indicated. Linear mixed effects models using replicate (1-5) as a random factor and fitted to each of the three phenotypes indicated that in all three phenotypes on average declined with relatedness. The rank of the coefficients was always High R, Mid R, Low R. A common observation in microbial cooperation is that when relatedness is lower, breakdown of cooperation is caused by cheats who exploit the cooperative signalling of others. Consistent with this we found that not only was growth and signal gene expression lower, they were also more variable in the lower relatedness treatment relative to the

others (Fig. 1d, 1f; $F_{1,13}$ = 10.9, 12.2, p = 0.002, 0.004). Although we found the same pattern with exoprotease production, it was non-significant, possibly due to one outlaying replicate which had a particularly low variance (Fig. 1e; $F_{1,13}$ = 2.96, p = 0.109). We then sequenced a random sample of three clones from each treatment in each replicate experiment (3 x 3 x 5 = 45 clones). The red data points represent the isolates that were sequenced. Single nucleotide polymorphisms in clonal isolates mapped to three known QS genes (*rsaL*, *vfr* and *lasI*) in three populations. The text above the panels indicate the gene that was mutated in that population and the arrows beside data point indicate which isolates had that mutation. All three QS mutations identified coincide with loss of QS activity (signal and protease production). The *rsaL* and the *lasI* mutations also coincide with loss of fitness in monoculture however the *vfr* mutation did not coincide with a loss in fitness. In addition to this, we sequenced clones that had lost the QS phenotypes but did not harbour mutations in known QS genes. Overall we conclude that many different genotypic routes can cause the phenotypes observed. For example in the case of *lasI* this could be a loss of function mutation however *rsaL* is a homeostatic inhibitor of the *lasIR* QS system. This means that potentially the mutation we observed in *rsaL* actually enhances its activity, thus reducing the overall QS activity of that clone.

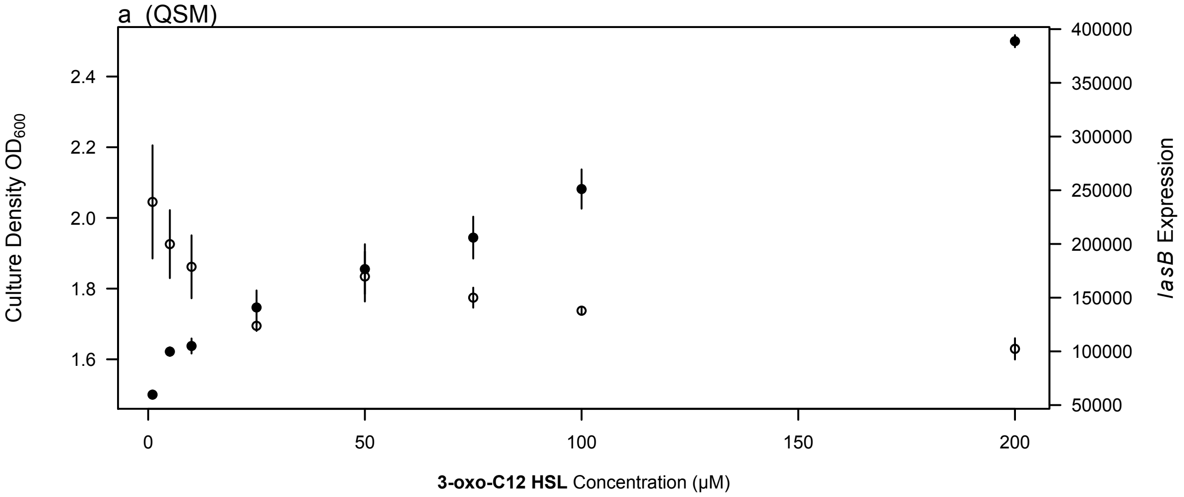

Figure S5. *Varying the cost:benefit ratio of signalling.* The effect of adding signal molecules on growth and *lasB* (codes for elastase) expression in a signal negative (*lasI*) mutant of the PAO1 wild type grown in QSM. All values are shown as a proportion of a treatment with no addition of signal. Manipulating QS activity by adding signal results in a fitness cost. There is a significant positive relationship between signal concentration and *lasB* expression (Filled circles, $F_{1,6} = 131.8$, $p < 0.001$). There is a significant negative relationship between growth (open circles) and signal concentration ($F_{1,6} = 131.8$, $p < 0.001$). We conclude that when the optimal level of QS output is exceeded in QSM there is a net cost, likely to impose a selective pressure in the long term evolutionary experiments.

Table S1. List of SNPs from individual isolates.
